# A phase 1/2 trial to assess safety and efficacy of a vaporized 5-methoxy-N,N-dimethyltryptamine formulation (GH001) in patients with treatment-resistant depression

**DOI:** 10.3389/fpsyt.2023.1133414

**Published:** 2023-06-20

**Authors:** Johannes T. Reckweg, Cees J. van Leeuwen, Cécile Henquet, Therese van Amelsvoort, Eef L. Theunissen, Natasha L. Mason, Riccardo Paci, Theis H. Terwey, Johannes G. Ramaekers

**Affiliations:** ^1^Department of Neuropsychology and Psychopharmacology, Faculty of Psychology and Neuroscience, Maastricht University, Maastricht, Netherlands; ^2^Department of Psychiatry and Neuropsychology, Faculty of Health, Medicine and Life Sciences, Maastricht University, Maastricht, Netherlands; ^3^GH Research, Dublin, Ireland

**Keywords:** psychedelics, 5-MeO-DMT, treatment-resistant depression, individualized dosing, clinical trial

## Abstract

**Background:**

Treatment-resistant depression (TRD) is a substantial public health burden, but current treatments have limited effectiveness. The aim was to investigate the safety and potential antidepressant effects of the serotonergic psychedelic drug 5-MeO-DMT in a vaporized formulation (GH001) in adult patients with TRD.

**Methods:**

The Phase 1 part (*n* = 8) of the trial investigated two single dose levels of GH001 (12 mg, 18 mg) with a primary endpoint of safety, and the Phase 2 part (*n* = 8) investigated an individualized dosing regimen (IDR) with up to three increasing doses of GH001 (6 mg, 12 mg, and 18 mg) within a single day, with a primary endpoint of efficacy, as assessed by the proportion of patients in remission (MADRS ≤ 10) on day 7.

**Results:**

Administration of GH001 via inhalation was well tolerated. The proportion of patients in remission (MADRS ≤ 10) at day 7 was 2/4 (50%) and 1/4 (25%) in the 12 mg and 18 mg groups of Phase 1, respectively, and 7/8 (87.5%) in the IDR group of Phase 2, meeting its primary endpoint (*p* < 0.0001). All remissions were observed from day 1, with 6/10 remissions observed from 2 h. The mean MADRS change from baseline to day 7 was −21.0 (−65%) and − 12.5 (−40%) for the 12 and 18 mg groups, respectively, and − 24.4 (−76%) for the IDR.

**Conclusion:**

Administration of GH001 to a cohort of 16 patients with TRD was well tolerated and provided potent and ultra-rapid antidepressant effects. Individualized dosing with up to three doses of GH001 on a single day was superior to single dose administration.

**Clinical Trial registration**: Clinicaltrials.gov Identifier NCT04698603.

## Introduction

5-Methoxy-N,N-dimethyltryptamine (5-MeO-DMT) is a serotonergic psychedelic from the tryptamine class, that primarily acts as an agonist at the 5-HT1A and 5-HT2A receptors (1, 2), and that, when delivered via pulmonary inhalation, has a rapid onset (about 5–10 s) and a short duration (about 5–30 min) of psychoactive effects (2–5). 5-MeO-DMT is a naturally-occurring substance, and it has a long history of use in naturalistic contexts, where its ability to induce altered states of consciousness (4, 6), often described as ego-dissolution and feelings of unity and connectedness with the universe, has been applied for spiritual or self-exploratory purposes (2, 7). Additionally, observational studies and a web-based survey on the naturalistic or recreational use of toad venom containing 5-MeO-DMT or synthetic 5-MeO-DMT have described subjective improvements in participant-reported measures of satisfaction with life and psychological well-being in people without an underlying mental health condition and reductions of depressive symptoms in people with self-reported depression (5, 8, 9). Further, antidepressant properties of other tryptamines such as psilocybin and DMT have been suggested in clinical populations in clinical trials (10–12). It is, however, unknown if 5-MeO-DMT might elicit therapeutic effects in mental disorders, and which doses of 5-MeO-DMT are required to occasion a therapeutic outcome.

A promising target indication for psychedelic treatment (13, 14) is treatment-resistant depression (TRD) which occurs in approximately 30 to 60% of patients with major depressive disorder (15, 16). The range is due to a lack of consensus regarding the definition for TRD. In clinical trials, an inadequate therapeutic response to at least one pharmacotherapy, one pharmacotherapy and one psychotherapy, or two pharmacotherapies within the same depressive episode has been used (17). Novel treatment options for TRD are desired, as conventional treatments appear to fail. Serotonergic psychedelics could potentially address this unmet need (14).

This study aimed to investigate the safety and efficacy of GH001, a proprietary vaporized 5-MeO-DMT formulation (GH Research, Dublin, Ireland) in patients with TRD. In the Phase 1 part of the study, TRD patients received single doses of 12 mg or 18 mg of GH001, and in the Phase 2 part, patients received an individualized dosing regimen (IDR) of up to three increasing doses of 6 mg, 12 mg, and 18 mg of GH001 within a single day. The IDR was designed (7) to control the significant inter-personal dose–response variability, which has been described for 5-MeO-DMT (8) and other serotonergic psychedelics (18, 19). The IDR is enabled by the short half-life of GH001, by its short duration of psychoactive effects and by its lack of tachyphylaxis. The short duration of psychoactive effects of GH001, together with their ineffable nature, also facilitates administration without specific psychotherapeutic interventions (preparation, guided treatment session, integration) as an integrated part of the therapeutic modality, as often done in other psychedelic development programs with psychedelic drugs inducing prolonged psychoactive effects. In the GH001 program, preparation and support is provided as part of standard medical care, and consists of standard informed consent, provision of a comfortable dosing environment, and availability of medical and psychological support throughout the study.

## Methods

The study was conducted at the Faculty of Psychology and Neuroscience, Maastricht University, The Netherlands. The study was approved by the Dutch Central Committee on Research Involving Human Subjects (CCMO) and the Medical Ethics Committee of the Academic Hospital of Maastricht and Maastricht University and conducted according to the principles of Good Clinical Practice (GCP) and the code of ethics on human experimentation established by the declaration of Helsinki (1964) and amended in Fortaleza (2013). The study was registered in the Dutch CCMO-register (NL70411.068.19), EudraCT (2018-004208-20), and clinicaltrials.gov (NCT04698603).

### Patients

**Figure 1:**
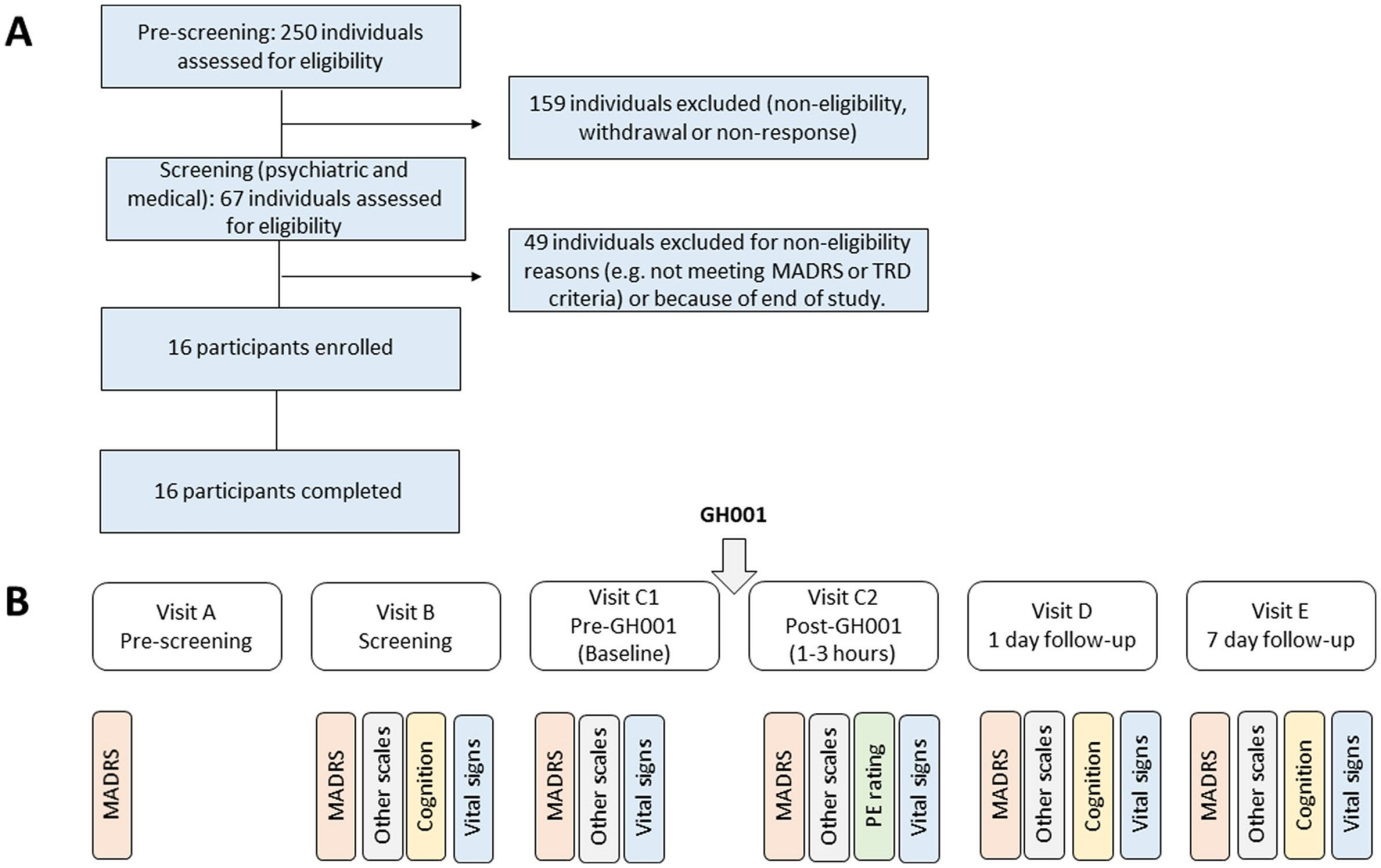
Upper panel **(A)** shows a diagram of participant flow. The lower panel **(B)** shows the flow of study assessments from pre-screening (Visit A) through Screening (Visit B), Baseline (Visit C1), after administration (Visit C2), 1 day follow-up (Visit D) to the 7 day follow-up (Visit E). Aside from the Montgomery-Åsberg Depression Rating Scale (MADRS), other scales included C-SSRS, BPRS, and CADSS, while the latter was limited to visits **(C)** pre and post, D and E. Vital signs included measures of heart rate and blood pressure as detailed in the Supplementary appendix. Measures of cognition included the PVT and DSST. All assessments at Visit C2 were taken between 1–3 h (D0-H2) after administration of GH001, as detailed in the Supplementary appendix. The peak experience (PE) rating was assessed through the Peak Experience Scale.

Participants were recruited through social media and search engines, flyers, recruitment agencies and psychological and psychiatric practices and institutes throughout the Netherlands and Belgium. Participants needed to fulfill the study criteria for TRD as confirmed by a clinical psychologist or psychiatrist, including meeting the diagnostic criteria according to the Diagnostic and Statistical Manual of Mental Disorders (DSM-5) for single-episode major depressive disorder (MDD) or recurrent MDD without psychotic features as confirmed by the Mini-International Neuropsychiatric Interview (MINI) (20); meeting criteria for a “valid” current major depressive episode based upon the Massachusetts General Hospital (MGH) SAFER criteria interview; (21) having a score of 28 or higher on the Montgomery-Åsberg Depression Rating Scale (MADRS) (22) at screening (and no more than a 20% decrease between the screening and the administration day); and a score of 4 or higher on the Patient’s Global Impression – Severity scale (PGI-S) (23, 24) for depression. Furthermore, participants needed to have an inadequate response (at most a minimal improvement of depressive symptoms) to at least two adequate courses of pharmacological therapy or one adequate course of pharmacological therapy and at least one adequate course of evidence-based psychotherapy within the current episode of depression. Adequacy of treatments was assessed with the Antidepressant Treatment History Form – Short Form (ATHF-SF) (25). Exclusion criteria included a previous or current diagnosis of a psychotic disorder or MDD with psychotic features, or bipolar disorder (or an immediate family history of the same). Additionally, participants with obsessive compulsive disorder, autism spectrum disorder, borderline personality disorder, clinically significant intellectual disability, or any other psychiatric comorbidity that rendered the participant unsuitable for the study or risk of suicidality as assessed by a clinical psychologist or psychiatrist, as well as any significant medical contraindication as assessed by a medical doctor were excluded. Participants that previously experienced a significant adverse reaction or demonstrated non-response of depressive symptoms to a psychedelic or dissociative drug were also excluded. Participants using antidepressants or other psychoactive compounds at screening followed a tapering off and washout procedure prior to study entrance. Participants gave written informed consent and received standard monetary compensation for their participation in the study. Participant characteristics such as age, sex, and race were self-reported. An overview of the flow of patient recruitment is presented in Figure 1.

### Design

The Phase 1 part of the study was designed as an open-label, single-arm, single-dose study with two dose groups. The first group (*n* = 4) received 12 mg of GH001, and the second group (*n* = 4) received 18 mg of GH001. The Phase 2 part was an open-label, single-arm study employing the IDR. Participants in this part (*n* = 8) received up to three increasing doses of GH001 (6 mg, 12 mg, and 18 mg) on a single day, spaced 3 h apart. The second and third doses were only administered in the event that the patient did not achieve a peak experience (PE) at the previously administered dose (7), if the previously administered dose was safe and well tolerated, and if both the patient and the medical doctor agreed. The choice of PE as a marker to guide the dose increase in the IDR is based on the hypothesis that, in the context of 5-MeO-DMT therapy, the intensity of the psychedelic experience correlates with clinical efficacy. Clinical trials with other serotonergic psychedelics had previously suggested that the intensity of the acute psychedelic experience is the main predictive factor of clinical response (26). The achievement of a PE was evaluated using a proprietary questionnaire, the PE Scale, which averages answers scored by the subject from 0 to 100 on a visual analogue scale for three parameters of the experience: intensity, feelings of loss of control, and profoundness (7). A PE is determined to have been achieved if the average score across these three parameters is at least 75. Participants were aware that all doses were active, however they were not informed about the actual identity of the study drug or dose to avoid participant bias through expectancy effects. Participants were informed about the identity of the substance and the dose(s) they received after completion of the study. Additional psychoactive effect assessments (Mystical Experience Questionnaire, Challenging Experience Questionnaire, 5-Dimensional Altered States of Consciousness Rating Scale) were performed. The results of those measures will be shared in a separate publication.

For both parts, the study consisted of 5 visits. During the first appointment (Visit A), a telephone suitability screening was conducted by a researcher to evaluate preliminary suitability of the participant. The second visit (Visit B) consisted of a medical screening in person and a psychiatric screening by a psychiatrist or clinical psychologist during a video or in-person meeting. GH001 was administered during Visit C. Follow-up meetings were scheduled at 1 day after the administration (Visit D) and at 7 days (range 6–8 days) after the administration (Visit E). No specific psychotherapeutic interventions, besides interactions for the screening and outcome assessments, were included at any of the visits. A study safety group (SSG), which included independent experts, evaluated the available safety data, data on psychiatric measures, and cognitive data to evaluate the safety and tolerability of the administered doses of GH001 after the Phase 1 part and the Phase 2 part of the study. A summary of the study design from pre-screening to the 7 day follow-up visit is provided in Figure 1.

### Study treatment

GH001 (GH Research, Dublin, Ireland) is an investigational drug product based on a proprietary formulation of synthetic, high purity, GMP pharmaceutical grade 5-MeO-DMT for administration via inhalation. GH001 was administered after a standardized vaporization procedure using the Volcano Medic Vaporization System (Storz and Bickel, Germany), approved in Europe, Australia, and Canada for medical use with cannabinoids (27–29). The device consists of a hot air generator, which facilitates formation of an aerosol from GH001, and a detachable valve balloon from which the aerosol is inhaled by the participant with a single breath. After inhalation, participants were instructed to hold their breath for 10 s before exhaling.

### Outcome measures

The primary endpoint of the Phase 1 part of the study was to assess the safety and tolerability of GH001 administered via inhalation after vaporization, as evaluated by a panel of measures: adverse event reporting, safety laboratory analyses, vital sign measures, electrocardiogram (ECG), psychiatric symptom measures [Brief Psychiatric Rating Scale (BPRS) (30), Columbia Suicide Severity Rating Scale (C-SSRS) (31), Clinician Administered Dissociative States Scale (CADSS) (32)], and measures of cognitive function [Psychomotor Vigilance Task (PVT) (33), Digit Symbol Substitution Task (DSST)] (34). Descriptions and results of these outcome measures and timing of assessment are provided in Supplementary material.

The primary endpoint of the Phase 2 part of the study was to assess the effect of GH001 on the severity of depression, as evaluated by the proportion of patients in remission (MADRS ≤ 10) at 7 days after dosing. The MADRS scale is a 10-item diagnostic questionnaire used to measure the severity of depressive episodes in patients with mood disorders (35). The overall score ranges from 0 to 60. MADRS assessments were performed at screening, at baseline before dosing of GH001, and at 2 h, 1 day and 7 days after dosing. The recall period for MADRS at screening and baseline comprised the previous 7 days, while the recall period at 2 h, 1 day and 7 days after dosing spanned from the time point when the acute psychedelic effects after dosing had subsided, to the assessment time point. At the 2 h time point, the sleep item was not evaluated. Instead, the pre-dose MADRS score for the sleep item recorded at baseline before dosing was carried forward, as similarly applied by Singh et al. (36). All MADRS assessments were performed remotely by a psychiatrist or clinical psychologist who did not witness the dose administration and was not involved in patient care. Safety and tolerability was a key secondary endpoint of the Phase 2 part of the study.

Secondary endpoints for both parts of the study included the mean MADRS change from baseline at 2 h, 1 day, and 7 days after dosing, and the proportion of patients in response (≥50% reduction from baseline in MADRS total score) at 7 days after dosing.

### Adverse events

Adverse events (AEs) were recorded from enrollment in the study after Visit B until completion of the study at Visit E. AEs were followed up until they resolved or were deemed no longer clinically significant. MedDRA version 22 was used for the coding of the AEs.

### Statistics

No formal sample size calculation was performed for the Phase 1 part of the study, but a sample size of 4 in each dose group was deemed sufficient to provide initial information on dose-related safety, efficacy, and psychedelic effects of GH001 to support the Phase 2 part of the study.

The sample size of 8 for assessment of the primary endpoint of the Phase 2 part of the study was calculated to achieve at least 90% power for a one-sided null hypothesis assuming a remission probability ≤1% and an assumed true remission probability of 50%, tested by an exact binomial test with one-sided significance level *α* = 0.025. The actual power for rejecting the null hypothesis was approximately 96%. A two-sided exact mid-*p* 95% confidence interval for the remission probability was also provided.

The secondary endpoints of mean change in MADRS total score from baseline to 2 h, 1 day, and 7 days after dosing were evaluated by a paired *t*-test comparing the mean MADRS total score at the respective time point with the mean MADRS total score at baseline, each time point being evaluated separately.

The components of the safety endpoint of both parts of the study (primary endpoint of the Phase 1 part and secondary endpoint of the Phase 2 part) were summarized descriptively for analysis by the SSG, which then provided its conclusion to the sponsor.

All analyses were carried out using IBM SPSS Statistics for Windows Version 28.0.1.

## Results

The study comprised 16 participants (7 females, 9 males), aged 21 to 51 years (median = 29.5). All participants were white and no differences between gender identity and sex assigned at birth were reported. Demographic data is summarised in Table 1. Participants were enrolled between November 2019 and September 2021, with a break in recruitment between March 2020 and June 2020 due to the COVID-19 pandemic.

**Table 1:** Demographics of study participants.

Characteristic	Phase 1 part		Phase 2 part
	12 mg (*n* = 4)	18 mg (*n* = 4)	IDR (*n* = 8)
Age, median (range), *y*	32 (24–51)	28.5 (21–50)	33.5 (21–47)
Female sex (%)	2 (50)	2 (50)	3 (37.5)
Time since initial diagnosis, median (range), *y*	11.5 (4–30)	12 (7–27)	9.5 (1–27)
Time in current episode, median (range), *mo*	42 (7–94)	54 (24–84)	46 (20–72)
Total adequate antidepressive treatment courses in current episode^a^, median (range)	2 (2–3)	2 (2–3)	2 (2–4)
Baseline MADRS, median (range)	33 (31–34)	32.5 (29–34)	32 (28–35)
Number (%) of participants tapered off antidepressants	2 (50)	1 (25)	2 (25)

a As assessed by the Antidepressant Treatment History Form – Short Form (ATHF-SF).

**Figure 2:**
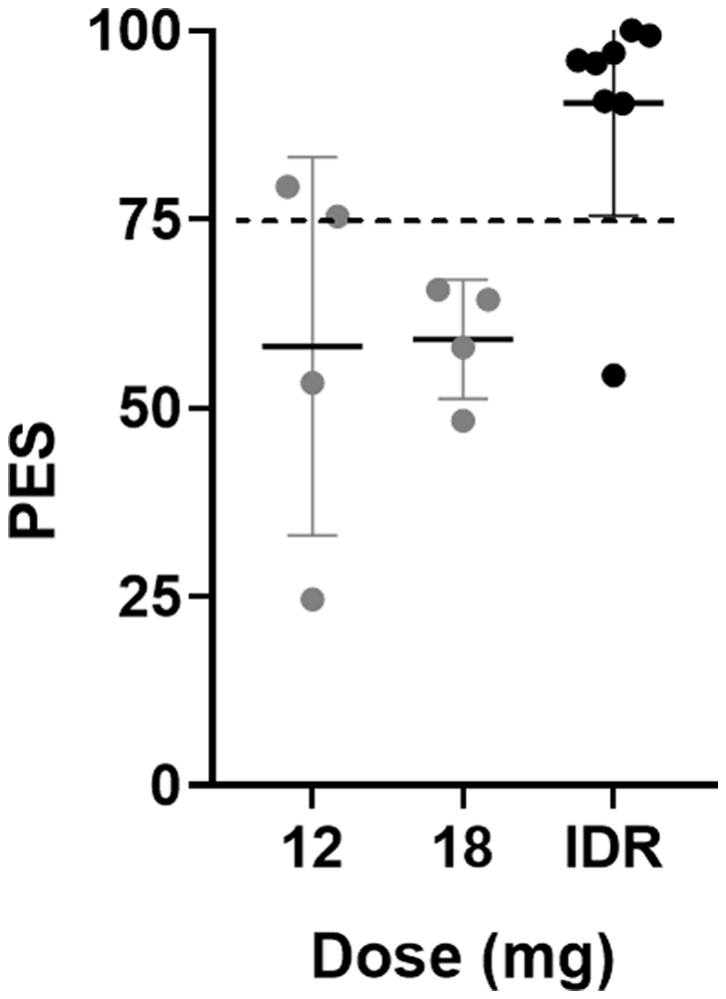
Mean (SE) and individual retrospective ratings of the acute psychedelic experience assessed with the Peak Experience Scale (PES) after single doses of 12 and 18 mg of GH001 and after the individualized dosing regimen (IDR).

Mean (SE) and individual ratings of the Peak Experience Scale (PES) are shown in Figure 2. In the Phase 1 part, 2 out of 4 patients achieved a PE (i.e., PES rating ≥ 75) in the 12 mg dose group, and 0 out of 4 patients achieved a PE in the 18 mg dose group. In the Phase 2 part, applying the IDR, 7 out of 8 patients achieved a PE, whereby 6 patients achieved a PE after the second administration (6 mg + 12 mg), and one patient achieved a PE after the third administration (6 mg + 12 mg + 18 mg). The intensity of the psychoactive effects increased with increasing dosage amounts of the IDR in the Phase 2 part, and at the maximum individual dose level, the mean PE total score was higher than in the single dose groups of the Phase 1 part.

**Figure 3:**
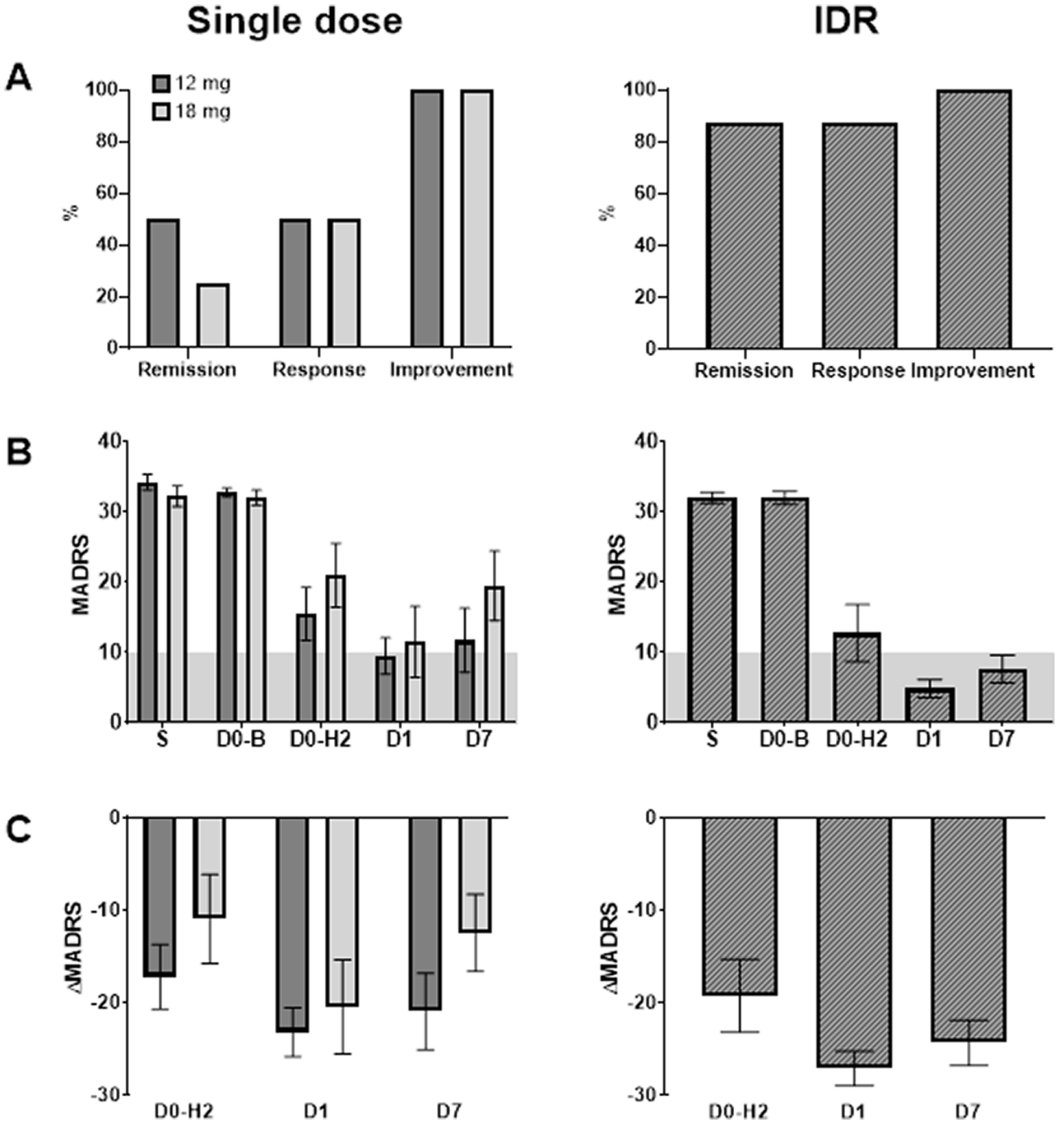
Panel **(A)** shows remission, response and improvement rates after single doses of GH001 and after an individualized dosing regimen (IDR) of GH001. Panel **(B)** shows mean (SE) of the Montgomery-Åsberg Depression Rating Scale (MADRS) ratings at screening (S), at baseline before dosing (D0-B), at 2 h after dosing (D0-H2), and at 1 (D1) and 7 (D7) days follow-up in the Phase 1 part (single dose) and the Phase 2 part (IDR). Grey planes indicate remission as indicated by MADRS ≤ 10. Panel **(C)** shows mean (SE) MADRS change from baseline at D0-H2, D1, and D7.

Mean (SE) MADRS ratings in the Phase 1 and Phase 2 parts of the study are shown in Figure 3. The proportion of patients with MADRS remission (MADRS ≤ 10) at day 7 was 2 out of 4 (50%) and 1 out of 4 (25%) in the 12 mg and 18 mg groups in the Phase 1 part, respectively, and 7 out of 8 (87.5%) in the IDR group in the Phase 2 part, meeting its primary endpoint (remission probability = 0.875; 95% CI = 0.473–0.997; Mid-*p* 95% CI = 0.520–0.994; *p* < 0.0001). Of the 10 patients in the study who had a remission at day 7, 8 achieved a PE. Of the 9 patients in the study who achieved a PE, 8 had a remission at day 7, whereby the 1 patient with a PE but no remission was a patient in the Phase 2 part of the study who had received all 3 GH001 doses, and thus had maximized the potential of the IDR. Of the 7 patients in the study who did not achieve a PE, 2 had a remission at day 7. Of the 10 remissions observed at day 7, all remissions were observed from day 1, with 6 of those 10 remissions observed from 2 h after the last dose. The mean MADRS change from baseline to day 7 was −21.0 (−65%) and − 12.5 (−40%) for the 12 and 18 mg groups, respectively, and −24.4 (−76%) for the IDR. Paired *t*-tests revealed a significant decrease in MADRS ratings at 2 h (*t* = −4.71; *p* = 0.0022), 1 day (*t* = −8.08; *p* < 0.0001), and 7 days (*t* = −5.31; *p* = 0.0011) after single dose administrations in the Phase 1 part, and at 2 h (*t* = −4.88; *p* = 0.0018), 1 day (*t* = −14.54; *p* < 0.0001), and 7 days (*t* = −9.98; *p* < 0.0001) after administration of the IDR in Part B. The time since initial diagnosis, the time in the current episode, the total number of antidepressive treatment courses in the current episode, and the baseline severity of depression did not correlate with the rate of MADRS remissions at day 7 or the mean MADRS change from baseline to day 7.

The assessment of the BPRS, while formally a safety assessment, revealed a strong reduction of overall psychiatric symptoms after administration of GH001 throughout the treatment week. The assessments of the C-SSRS and the CADSS, which were also included as safety endpoints, did not show any clinically significant change at any post-dose assessment as compared to their values at baseline. PVT and DSST were included in order to capture potential negative effects on cognition and did not show any impairment of cognitive function. Further, no clinically significant changes in vital parameters, ECG and safety laboratory analyses were observed. A summary of these measures is provided in Supplementary material.

A summary of adverse drug reactions (ADRs) is listed in Table 2, all of them being mild or moderate and resolving spontaneously. No Serious Adverse Events (SAEs) were reported. In their assessment of the overall safety data in the context of the safety endpoints, the SSG concluded that administration of GH001 via inhalation was safe and well tolerated for the investigated single dose levels of the Phase 1 part and for the IDR of the Phase 2 part of the study and considered the endpoints met. A summary of mean (SE) systolic and diastolic blood pressure and heart rate is given in Supplementary material.

**Table 2:** Adverse Drug Reactions (ADRs), i.e., treatment-emergent adverse events reported with a relationship to the investigational product as definite, probable, or possible, by MedDRA System Organ Class and Preferred Term.

Severity	System organ class preferred term	Phase 1 part		Phase 2 part
		12 mg(*n* = 4)	18 mg(*n* = 4)	6 + 12 +18 mg (IDR) (*n* = 8)
Mild	*Gastrointestinal disorders*			
	Abdominal discomfort	–	–	1
	*General disorders and administration site conditions*			
	Feeling abnormal	1	1	–
	*Musculoskeletal and connective tissue disorders*			
	Muscle spasms	–	1	–
	Muscle discomfort	–	–	1
	*Nervous system disorders*			
	Dizziness	1	–	–
	Headache	2	1	3
	Paraesthesia	–	–	1
	Sensory disturbance	–	–	3
	*Psychiatric disorders*			
	Anxiety	–	–	2
	Flashback	1	1	2
Moderate	*Gastrointestinal disorders*			
	Nausea	–	–	2
	*Psychiatric disorders*			
	Depressive symptom	–	–	1

## Discussion

The twofold aim of this study was to assess safety and efficacy of single-day dosing of a GH001 formulation for inhaled delivery of 5-MeO-DMT in patients with TRD. In the Phase 1 part of the trial, patients with TRD received a single dose of GH001 (either 12 or 18 mg) whereas in the Phase 2 part, a flexible IDR was applied to control the inter-personal dose variability commonly observed with administration of serotonergic agents, thereby aiming to optimize the therapeutic benefit, while at the same time avoiding unnecessarily high doses. Applying the IDR, 7 out of 8 patients (87.5%) achieved remission (MADRS ≤ 10) at day 7 after GH001 dosing with a mean MADRS reduction vs. baseline of −24.4 (*p* < 0.0001). This was superior to the outcome achieved with single 12 mg and 18 mg doses of GH001 in the Phase 1 part of the GH001-TRD-102 trial, where 2 out of 4 patients (50%) and 1 out of 4 patients (25%) achieved a remission (MADRS ≤ 10) at day 7 after dosing, with mean MADRS reductions vs. baseline of −21.0 and −12.5, respectively.

The antidepressant effect of GH001 occurred rapidly after administration and all remissions were observed from day 1, with 6 of 10 remissions already observed from 2 h. Even with the small sample size, these findings suggest that GH001 can exert a fast and significant reduction in depressive symptoms that can culminate in a full remission throughout 1 week after dosing. According to FDA draft guidance for industry “Major Depressive Disorder: Developing Drugs for Treatment,” a 1 week endpoint is an appropriate primary efficacy endpoint for rapid-acting antidepressants (37).

Peak experiences (i.e., PES ≥ 75) were recorded in 7 out of 8 patients in the IDR group and in 2 out of 8 patients in the single dose group. This indicates that peak psychoactive experiences are more likely to be achieved after the IDR regimen as compared to single dose administration of GH001. This finding is in line with a previous study in healthy volunteers that also reported more peak experiences in the IDR group as compared to the single dose groups (7). Importantly, the proportion of patients in remission that achieved a PE was 8 out of 10 and the proportion of patients with a PE that achieved a remission was 8 out of 9, while the proportion of patients without a PE that achieved a remission was only 2 out of 7. This supports that the magnitude of a psychedelic experience is a strong predictor of a positive therapeutic response in patients suffering from depression (10, 26, 38). No patient with a PE and less than 3 doses failed to achieve a remission, validating the IDR from a clinical dosing targeting perspective.

In this trial, no safety signals were observed in terms of any severe adverse effects, and in terms of any of the safety laboratory analyses, vital signs, psychiatric safety assessments or measures of cognitive function. In fact, assessment of the BPRS, while formally a safety assessment, revealed a strong reduction of overall psychiatric symptoms after administration of GH001 throughout the treatment week. These results are in line with safety data from a previous trial with GH001 in healthy volunteers (7), and further attest to the safety profile of the GH001 dosing approach, which is delivered in an outpatient setting with standard supportive care, but without extensive requirements for the therapeutic environment and without specific psychotherapeutic interventions before, during and after dosing, as done in other psychedelic development programs. In conclusion, administration of the inhaled GH001 formulation of 5-MeO-DMT in an outpatient setting to a cohort of 16 patients with TRD was well tolerated and provided potent and ultra-rapid antidepressant effects. Individualized dosing with up to three doses on a single day was superior to single dose administration. The finding of an antidepressant effect of GH001 in this open-label study warrants further clinical research to confirm efficacy and safety in a larger patient population as part of a randomized, controlled clinical trial.

## Data availability statement

The datasets presented in this article are not readily available because data are proprietary to GH Research. The datasets presented in this article are not readily available to protect proprietary information. Requests to access the datasets should be directed to clinicaltrials@ghres.com.

## Ethics statement

The studies involving human participants were reviewed and approved by Medical Ethics Review Committee azM/UM. The patients/participants provided their written informed consent to participate in this study.

## Author contributions

TT and JRa were involved in designing the study. JRe, JRa, NM, TA, CH, RP, and CL were involved in data collection and study logistics. JRe, JRa, and TT were involved with data analysis, interpretation, and visualization. All authors contributed to the article and approved the submitted version.

## Conflict of interest

JRe and JRa are scientific consultants to GH Research. TT is an employee and shareholder of GH Research. The authors declare that this study received funding from GH Research. The funder had the following involvement in the study: study design, data analysis and preparation of the manuscript.

The remaining authors declare that the research was conducted in the absence of any commercial or financial relationships that could be construed as a potential conflict of interest.

## Publisher’s note

All claims expressed in this article are solely those of the authors and do not necessarily represent those of their affiliated organizations, or those of the publisher, the editors and the reviewers. Any product that may be evaluated in this article, or claim that may be made by its manufacturer, is not guaranteed or endorsed by the publisher.
