# Supplementary material for: A phase 1/2 trial to assess safety and efficacy of a vaporized 5-methoxy-N,N-dimethyltryptamine formulation (GH001) in patients with treatment-resistant depression
Source: Front Psychiatry. 2023 Jun 20;14:1133414. doi: 10.3389/fpsyt.2023.1133414 (PMC10319409; doi:10.3389/fpsyt.2023.1133414)
Supplement: Supplementary file 1 [file Data_Sheet_1.pdf]

SUPPLEMENT

A Phase 1/2 Trial to Assess Safety and Efficacy of a Vaporized 5-Methoxy-N,N-Dimethyltryptamine Formulation (GH001) in Patients with Treatment-Resistant Depression

Johannes T. Reckweg[1*], Cees J. van Leeuwen[1], Cécile Henquet[2], Therese van Amelsvoort[2], Eef L. Theunissen[1], Natasha L. Mason[1], Riccardo Paci[1], Theis H. Terwey[3], Johannes G. Ramaekers[1*]

[1] Department of Neuropsychology and Psychopharmacology, Faculty of Psychology and Neuroscience, Maastricht University, Maastricht, The Netherlands

[2] Department of Psychiatry and Neuropsychology, Faculty of Health, Medicine and Life Sciences, Maastricht University, The Netherlands

[3] GH Research, Dublin, Ireland

Correspondence: j.ramaekers@maastrichtuniversity.nl and johannes.reckweg@maastrichtuniversity.nl

METHODS

Procedure

After receipt of a completed informed consent form from candidate participants, a structured telephone interview was conducted with the participant by a trained investigator at visit A. This call was centered around the inclusion and exclusion criteria to perform a preliminary evaluation of the patient's eligibility. In the case of a positive evaluation, the screening visit, visit B, was scheduled. The participant was invited to Maastricht University for a medical screening and a practice session of the GH001 inhalation procedure. Additionally, an intake interview by a psychologist structured around the Brief Psychiatric Rating Scale (BPRS) and a psychiatric screening by a clinical psychologist or psychiatrist were conducted. These psychiatric measures were carried out either in person or via an online video call on another day, usually shortly prior to the in-person appointment of visit B, depending on the availability of the patient and staff. If the participant was deemed eligible, baseline assessments of cognitive function tests were taken. If appropriate, a schedule for tapering off any current antidepressant medication was initiated. The duration of the tapering was chosen per investigator discretion, the duration of additional wash-out after the last dose was 2 weeks (5 weeks in case of fluoxetine). Participants were allowed to continue ongoing psychotherapy but were not permitted to initiate a new psychotherapeutic treatment during participation in the study. Participants were also provided with a 24-hour contact phone number to reach the clinical team in case of questions, psychological difficulties, or medical problems during the whole study. At visit C, the test day, patients were asked to arrive fasted and well rested. Participants were not allowed to consume alcohol on the day prior to the test day or on the test day and were prohibited from smoking on the test day. Together with reconfirmation of participant eligibility, drug and alcohol tests were performed, as well as pregnancy tests for women. The participants then had another remote interview for baseline assessments of MADRS and C-SSRS with a clinical psychologist or psychiatrist who was not involved in dose administration or patient care and a conversation with a psychologist, who was part of

the study team, for assessment of the BPRS. Further baseline assessments for the CADSS, electrocardiogram (ECG), vital signs and standard safety laboratory tests were taken. A Caretaker 4 (Caretaker Medical EMEA, UK) device was installed on the patient for continuous remote monitoring of vital signs including continuous non-invasive, beat-by-beat blood pressure, heart rate, oxygen saturation, temperature, respiration rate, body temperature, and an ECG throughout the test day. Additional manual cuff-based measurements (Omron M6) of blood pressure and heart rate were done at 30 minutes before and at 1 and 3 hours after dosing with GH001, while participants were in a supine position after 4 minutes of rest, and these standard recordings formed the basis of the documented vital signs. Per protocol, the output of the Caretaker 4 system was only considered supportive, as movement artifacts during and after exposure to GH001 easily occurred and recalibration after change of posture would have been required. After the administration, the participant was asked to lie on a comfortable mattress. The participant was accompanied in the administration room by a medical doctor and at least one researcher for one hour. Afterwards, a meal was provided and the participant completed questionnaires assessing the psychedelic experience. Another remote psychiatric interview was conducted about 2 hours after the final administration for assessment of the MADRS and the C-SSRS, followed by assessments of the BPRS and CADSS and recording of ECG and collection of safety laboratory analyses, before the medical supervisor determined if the participant was fit to be discharged. The participant returned the next day for visit D, which consisted of another remote psychiatric interview, the conversation with a psychologist based on the BPRS, vital sign measurements, and assessments of cognitive functioning (DSST, PVT). The last study appointment, visit E, was performed 7 days (± 1 day) after the administration day and was identical to visit D, with the addition of blood samples for laboratory safety analyses. No specific psychotherapeutic interventions, besides interactions for the screening and outcome assessments, were planned at any of the visits.

Psychiatric safety assessments as part of the safety endpoint

Columbia Suicide Severity Rating Scale (C-SSRS)

The C-SSRS [1,2] is a detailed questionnaire assessing two main endpoints, suicidal behavior and suicidal ideation. The questionnaire was administered as an interview by a registered psychologist or psychiatrist at screening, at baseline, and at 3 hours, 1 day, and 7 days after administration of study drug. Each endpoint is comprised of five items, rated through a binary yes/no response. The score of each endpoint equals the number of yes-responses for that category. The total C-SSRS score (range: 0 – 10) is the sum of the two endpoint scores.

Clinician Administered Dissociative States Scale (CADSS)

The CADSS [3] comprises 19 subjective items, ranging from 0 'not at all' to 4 'extremely'. It is divided into 3 components: 1) depersonalization, 2) derealization and 3) amnesia. Summed together, these subscales form a total dissociative score (range: 0 – 76). The CADSS was specifically designed to be a standardized measure of present-state dissociative symptomatology. The CADSS was administered at baseline and at 3 hours, 1 day, and 7 days after administration of study drug.

Brief Psychiatric Rating Scale (BPRS)

The BPRS [4] was intended to screen for psychiatric symptoms in a structured fashion and was evaluated at screening, at baseline, at 3 hours after administration of study drug before the participant left the site (as part of the 'closing-out interview'), and at days 1 and 7 after administration of study drug). Each symptom was rated 1-7 and a total of 18 symptoms were scored (range: 18 – 126).

Psychomotor Vigilance Task (PVT)

The PVT [5] assessed the reaction time (RT) in response to a visual stimulus. The visual stimulus was a counter in the middle of a computer screen that started at random intervals between 2 and 10 seconds. The participant responded by pressing a button as soon as possible after the counter started. Duration of the task was 6 minutes. The process was used to measure sustained attention performance. Outcome measure was the number of attentional lapses (RT ≥ 500 msec). The PVT was administered at screening, and at 1 day and 7 days after administration of study drug.

Digit symbol substitution task (DSST)

The DSST [6] was a computerized version of the original paper and pencil test taken from the Wechsler Adult Intelligence Scale. The participant was shown an encoding scheme consisting of a row of squares at the top of the screen, wherein nine digits were randomly associated with particular symbols. The same symbols were presented in a fixed sequence at the bottom of the screen as a row of separate response buttons. The randomization procedure was chosen such that symbols never appeared at the same ordinal position within both rows. The encoding scheme and the response buttons remained visible while the participant was shown successive presentations of a single digit at the center of the screen. The task was to match each digit with a symbol from the encoding list and click the corresponding response button. The measured cognitive operations include attention, motor performance, and visuoperceptual functions. The percentage of digits correctly encoded among the total number of responses within 3 minutes was the performance measure. The DSST was administered at screening, and at 1 day and 7 days after administration of study drug.

Table S1. Mean (SE) Brief Psychiatric Rating Scale (BPRS) scores from screening to the 7-day follow-up visit.

Dose		Visit B<br>screening	Visit C1<br>pre GH001<br>(baseline)	Visit C2<br>post GH001<br>(3 hours)	Visit D<br>1 day follow-up	Visit E<br>7 day follow-up
12mg	Mean	44.5	45.3	31.3	22.5	22.3
	SE	1.50	1.44	2.78	2.33	2.66
18mg	Mean	35.0	32.5	24.0	26.8	25.5
	SE	3.11	2.47	2.74	4.11	4.01
IDR	Mean	32.4	32.4	22.0	20.0	20.1
	SE	1.50	1.40	1.49	0.57	0.67

Table S2. Mean (SE) rating of amnesia, depersonalization, derealization and their score as assessed with the CADSS at baseline and post GH001 administration.

Dose		Visit C1<br>pre GH001<br>(baseline)	Visit C2<br>post GH001<br>(3 hours)	Visit D<br>1 day follow-up	Visit E<br>7 day follow-up
Amnesia					
12mg	Mean	0.0	0.0	0.0	0.3
	SE	0.00	0.00	0.00	0.25
18mg	Mean	0.0	0.0	0.0	0.3
	SE	0.00	0.00	0.00	0.25
IDR	Mean	0.1	0.3	0.0	0.0
	SE	0.13	0.16	0.00	0.00
Depersonalization					
12mg	Mean	1.0	0.8	0.3	0.0
	SE	0.58	0.75	0.25	0.00
18mg	Mean	0.0	0.3	0.0	0.0
	SE	0.00	0.25	0.00	0.00
IDR	Mean	0.0	0.5	0.0	0.1
	SE	0.00	0.19	0.00	0.13
Derealization					

12mg	Mean	3.5	1.8	0.5	0.3
	SE	3.18	1.75	0.29	0.25
18mg	Mean	0.3	0.8	0.3	0.8
	SE	0.25	0.48	0.25	0.75
IDR	Mean	0.3	0.9	0.3	0.4
	SE	0.25	0.40	0.16	0.26
Total					
12mg	Mean	4.5	2.5	0.8	0.5
	SE	3.57	2.50	0.48	0.50
18mg	Mean	0.3	1.0	0.3	1.0
	SE	0.25	0.71	0.25	1.00
IDR	Mean	0.4	1.6	0.3	0.5
	SE	0.26	0.65	0.16	0.38

Table S3. Mean (SE) Columbia Suicide Severity Rating Scale (C-SSRS) total scores from screening to the 7-day follow-up visit.

Dose		Visit B screening	Visit C1 pre GH001 (baseline)	Visit C2 post GH001 (3 hours)	Visit D 1 day follow-up	Visit E 7 day follow-up
12mg	Mean	4.0	1.5	0.0	0.5	1.0
	SE	1.47	0.65	0.00	0.50	0.71
18mg	Mean	2.0	1.0	0.0	0.0	1.3
	SE	1.08	0.41	0.00	0.00	0.95
IDR	Mean	1.5	0.6	0.0	0.3	0.4
	SE	0.19	0.26	0.00	0.25	0.18

Table S4. Mean (SE) percentage correct substitutions in the Digit Symbol Substitution Task at screening and at the 1 and 7-day follow-up visit.

Dose		Visit B screening	Visit D 1 day follow-up	Visit E 7 day follow-up
12mg	Mean	97.530	99.468	98.960
	SE	1.7466	0.3094	0.6004
18mg	Mean	98.802	99.430	99.073
	SE	0.4588	0.3372	0.9267
IDR	Mean	98.708	98.691	99.279
	SE	0.5052	0.5359	0.3012

Table S5. Mean (SE) number of lapses in the Psychomotor Vigilance Task (PVT) at screening and at the 1 and the 7-day follow-up visit.

Dose		Visit B screening	Visit D 1 day follow-up	Visit E 7 day follow-up
12mg	Mean	8.8	0.8	0.5
	SE	5.42	0.75	0.50
18mg	Mean	0.5	0.5	0.0
	SE	0.29	0.50	0.00
IDR	Mean	0.6	0.6	0.0
	SE	0.32	0.26	0.00

Table S6. Mean (SE) heart rate from screening to the 7-day follow-up visit.

Dose		Visit B screening	Visit C pre dose 1 GH001 (baseline)	Visit C post dose 1 GH001 (1 hour)	Visit C pre dose 2 GH001	Visit C post dose 2 GH001 (1 hour)	Visit C pre dose 3 GH001	Visit C post dose 3 GH001 (1 hour)	Visit C post GH001 (3 hours)	Visit D 1 day follow-up	Visit E 7 day follow-up
12mg	Mean	65.3	68.8	70.5	-	-	-	-	59.0	66.8	67.8
	SE	7.97	1.75	3.18	-	-	-	-	2.80	6.49	6.36
18mg	Mean	75.3	72.3	73.3	-	-	-	-	67.8	76.0	87.0
	SE	3.61	8.27	6.49	-	-	-	-	2.56	4.10	10.07
IDR	Mean	73.0	71.5	69.5	71.6	77.4	69.5	68.5	76.5	83.6	77.0
	SE	3.74	4.65	3.22	3.13	3.74	0.50	3.50	4.30	3.65	3.66

Table S7. Mean (SE) systolic and diastolic blood pressure (mmHg) from screening to the 7-day follow-up visit.

Dose		Visit B screening	Visit C1 pre dose 1 GH001 (baseline)	Visit C2 post dose 1 GH001 (1 hour)	Visit C1 pre dose 2 GH001	Visit C2 post dose 2 GH001 (1 hour)	Visit C1 pre dose 3 GH001	Visit C2 post dose 3 GH001 (1 hour)	Visit C2 post GH001 (3 hours)	Visit D 1 day follow-up	Visit E 7 day follow-up
Systolic BP											
12mg	Mean	124.3	125.3	118.8	-	-	-	-	123.8	120.8	126.3
	SE	5.79	7.93	3.94	-	-	-	-	7.67	7.44	5.60
18mg	Mean	116.0	114.8	118.5	-	-	-	-	111.5	117.3	113.3
	SE	2.80	4.68	1.19	-	-	-	-	1.94	0.48	0.67
IDR	Mean	124.1	118.9	119.0	120.1	122.6	119.5	119.5	121.6	122.9	120.9
	SE	2.89	4.22	3.78	3.52	3.32	18.50	19.50	3.63	2.96	2.24
Diastolic BP											
12mg	Mean	72.8	77.3	75.5	-	-	-	-	72.0	76.5	79.0
	SE	2.59	1.31	2.02	-	-	-	-	2.94	2.53	2.71
18mg	Mean	74.5	72.5	75.8	-	-	-	-	68.8	72.8	77.7
	SE	2.06	3.57	1.70	-	-	-	-	2.02	5.50	4.06
IDR	Mean	75.3	78.1	78.3	72.9	76.3	72.5	75.0	74.0	75.9	76.1
	SE	2.27	1.82	2.23	1.61	1.52	2.50	0.00	1.69	1.83	1.16

Note: Except for temporary, non-clinically relevant increase in heart rate and blood pressure shortly after administration of GH001, no noteworthy changes were observed in the remote monitoring of vital signs with the Caretaker device, which included continuous non-invasive beat-by-beat blood pressure, heart rate, oxygen saturation, temperature, respiration rate, body temperature, and an ECG throughout the test day.

References

1. Brent DA, Greenhill LL, Compton S, et al. The Treatment of Adolescent Suicide Attempters study (TASA): predictors of suicidal events in an open treatment trial. *Journal of the American Academy of Child and Adolescent Psychiatry* 2009; **48**(10): 987-96.
2. US Food and Drug Administration. Guidance for Industry: Suicidality: Prospective Assessment of Occurrence in Clinical Trials, Draft Guidance. Rockville, MD: US Department of Health and Human Services; 2012.
3. Bremner JD, Krystal JH, Putnam FW, et al. Measurement of dissociative states with the clinician-administered dissociative states scale (CADSS). *Journal of Traumatic Stress: Official Publication of The International Society for Traumatic Stress Studies* 1998; **11**(1): 125-36.
4. Overall JE, Gorham DR. The Brief Psychiatric Rating Scale. *Psychological Reports* 1962; **10**(3): 799-812.
5. Lim J, Dinges DF. Sleep deprivation and vigilant attention. *Annals of the New York Academy of Sciences* 2008; **1129**(1): 305-22.
6. Royer FL, Janowitch L. Performance of process and reactive schizophrenics on a symbol-digit substitution task. *Perceptual and Motor Skills* 1973; **37**(1): 63-70.
